# A mental model of obesity: comparing expert and public perceptions in Ireland, the UK, and the US

**DOI:** 10.1038/s41366-026-02098-z

**Published:** 2026-06-05

**Authors:** Deirdre A. Robertson, Ylva Andersson, Ciarán Lavin, Peter D. Lunn

**Affiliations:** 1 Behavioural Research Unit, Economic and Social Research Institute, Dublin, Ireland; 2 School of Psychology, Trinity College Dublin, Dublin, Ireland; 3 Department of Social Psychology, Tilburg University, Tilburg, Netherlands; 4 Sustainable Energy Authority of Ireland, Dublin, Ireland; 5 Department of Economics, Trinity College Dublin, Dublin, Ireland

**Keywords:** Health policy, Public health, Disease prevention

## Abstract

**Objective:**

Despite scientific evidence on the environmental causes of obesity, policies that target these causes frequently face public opposition. This study investigates the mental model that the public hold about obesity, including perceptions of the causes, consequences and responsibility for obesity, alongside support for different preventive policies.

**Methods:**

*N* = 2400 members of the public undertook a cross-sectional online survey across 3 English-speaking countries with high obesity rates: Ireland, the UK and the US. We benchmarked public perceptions against *N* = 51 experts.

**Results:**

We found differences between countries and larger differences between the public and expert samples. The public assigned considerable blame and responsibility to individuals, while the expert sample focused more on societal and environmental causes. Although the public and expert samples concurred on the seriousness of the problem, the latter endorsed more radical policies, such as taxes and restrictions.

**Conclusions:**

Public health communications often focus on highlighting obesity as a public health problem, but our findings suggest that this message has been received. Instead, more work may be needed to challenge public beliefs about the causes of, and responsibility for, the obesity epidemic.

## Introduction

Since 1975, obesity rates have tripled worldwide [1]. Severe obesity is expected to double between 2020 and 2035 [2]. Obesity is defined as having excess weight to the extent that it impairs health [3]. It contributes to other non-communicable diseases including diabetes, metabolic syndrome, and liver disease [2]. Globally, obesity is responsible for 2.8 million deaths per year [1].

There are multiple causes of overweight and obesity including individual differences and biological factors, but the environment in which people live makes a substantive contribution. Massive changes to food systems, culture, economics and mechanisation of some jobs have resulted in more sedentary lifestyles and increased consumption [3, 4]. Since 1975, globally, the average number of daily calories available has risen by over 500; the equivalent of two personal size chocolate bars per person per day [5]. While there remains debate within medical science about which model best captures the pathogenesis of obesity [6], there is widespread agreement that the epidemic is driven by environmental changes, whether to the formulation, availability and volume of foods, or to exposure to chemicals that alter people’s energy regulation [7].

Public health policy can pursue stricter regulation of environmental factors, but obesity-related policies can be controversial. In 2012, New York regulated the size of sugary drinks to a maximum of 16 oz. Such was the opposition that the beverage industry sued the city and won in 2014 [8]. In Denmark, a tax on saturated fats was introduced for public health reasons in 2011 and repealed for fiscal reasons in 2012 [9]. Some policies, such as the sugar tax in the United Kingdom, have been implemented successfully, but not without opposition [10].

Support for, or opposition to, obesity-related policies can be driven by what people believe, mistakenly or otherwise, are the causes of obesity [11]. Members of the public can be unaware of how much the environment contributes to obesity, despite the scientific evidence [12]. Many people believe obesity is down to the individual, that people with obesity are lazy or lack willpower [11, 13–17]. This individual choice narrative tends to push people to support modest obesity policies, such as informational campaigns, but oppose stricter policies, such as taxes or restrictions [11, 15, 18, 19].

In the cognitive sciences, a mental model refers to someone’s internal representation of something [20]. People use their knowledge, perceptions, beliefs and assumptions to interpret information about specific phenomena and use this to guide their decisions. Understanding mental models of obesity could give insight into specific misperceptions and knowledge gaps that could be addressed to advance obesity prevention policies. This is the aim of this paper. We investigate beliefs, knowledge, perceptions and assumptions about the causes, consequences, seriousness and responsibility for the obesity epidemic, together with support for policies that might address it. This is the first study, to our knowledge, to undertake such a detailed exploration of the mental model of obesity and to compare it against expert views.

We do this in three countries. The obesity epidemic is global, but its trajectory has differed between countries. There can be variation in how populations of different countries perceive obesity [11, 21]. Cultural differences or exposure to the epidemic over different periods of time may influence mental models. We chose three developed countries with similar education levels and English as a first language (because translation of questions may change interpretation), but where rates of obesity had developed at different trajectories [22]: the United Kingdom (UK), Ireland and the United States (US). The US has had higher rates of obesity than the other two countries and an obesity epidemic for a longer period. As attitudes have had longer to form, people may be more attuned to the obesity epidemic and this might influence the mental model. Ireland and the UK are neighbouring countries, but the UK had higher rates of obesity than Ireland originally, after which Ireland has experienced a more rapid rise and surpassed the UK on some measures [23].

Finally, as there is no objective benchmark against which to test the accuracy of the beliefs we are measuring, we compare public views to those of a sample of experts in the field.

## Methods

The study consisted of an extensive, observational, cross-sectional survey undertaken in the three countries. (A related experiment designed to test pre-registered hypotheses is reported separately [24]. This paper reports exploratory analysis - we did not specify confirmatory hypotheses. The study was approved by the ESRI Research Ethics Committee. The research was performed in accordance with the Declaration of Helsinki. All participants gave informed consent. Pre-registration, data and analysis files are on Open Science Framework.^1^

### Participants

Participants took part online via laptop, phone, or tablet. They were told the purpose of the study was to assess public perceptions of obesity. Participants (*N* = 2400) were recruited from Ireland (*n* = 800), the UK (*n* = 800), and the US (*n* = 800). The UK and Irish samples were recruited from two market research companies^2^, the US sample was recruited through Prolific using the representative sampling feature. Participants were paid €3/£3 for participating in the 15-min study. A sample of obesity experts in Ireland and the UK was recruited through emails to universities, special interest groups, and public health organisations. An expert was defined as anyone with professional expertise in an area related to obesity working in Ireland or the UK. The experts were personally invited by the research team to participate. Invites targeted researchers who had published academic papers on obesity in the disciplines of public health, medicine, nutrition and physical activity, individuals working on obesity, nutrition or physical activity policies in government and healthcare workers specialising in obesity. Two expert groups working in obesity were asked to circulate to their members. A response rate thus cannot be calculated but the final number of experts who completed the survey was 51. Of the final expert sample, 33 were from Ireland, 18 were from the UK, 10% worked in government or policy, 31% in healthcare, 57% in research and 2% in other. The characteristics of the public samples is shown in Table 1.

**Table 1:** Sociodemographic breakdown of the public samples.

	Total sample	Ireland	UK	US
Age	48.0 (16.4)	50.2 (15.2)	48.0 (17.5)	45.8 (16.1)
Male	50.7%	53.8%	49.1%	49.2%
Degree+^a^	46.4%	34.8%	42%	62.5%
Social Grade				
ABC1	53.2%	41.8%	55.5%	62.4%
C2DE	43.1%	55.1%	44.4%	29.8%
F/Unsure	3.7%	3.1%	0.1%	7.9%
Employed	61.7%	61%	58%	66%
Employment sector				
None of these	85.2%	86.4%	84.8%	84.4%
Hospitality	3.5%	3.3%	2.6%	4.5%
Healthcare	6.9%	7%	7.3%	6.4%
Government	4.5%	3.4%	5.4%	4.8%
BMI (self-reported height and weight)				
<18.5 kg/m^2^	2.2%	1.8%	2.1%	2.8%
18.5 kg/m^2^ to <25 kg/m^2^	35.7%	33.1%	38%	35.9%
25 kg/m^2^ to <30 kg/m^2^	27.6%	29.1%	27.6%	26.1%
>30 kg/m^2^	34.5%	36%	32.3%	35.3%
Observations	2400	800	800	800

^a^Degree is defined as a bachelor’s degree or over in Ireland and the UK and an associate’s degree or higher in the US.

### Measures

Existing attitude scales do not distinguish causes of obesity from negative stereotypes of people with obesity [25]. Moreover, our aim was to compare perceptions of different types of causes directly (e.g. individual versus environmental). Given the lack of validated scales for this purpose, we designed multiple instruments to measure perceived causes and checked for consistent results across measures.

#### Perceived causes of the obesity epidemic

We measured perceived causes qualitatively and quantitatively. Participants completed the sentence “There is an obesity epidemic because…” in open text boxes. They could give one cause per box and submit up to ten. Two authors (DR, YA) coded these into four categories: individual choice or behaviour, physical/psychological/biological limitations and societal/obesogenic environment. Answers that could not be coded into one of these were listed as “ambiguous”.

Participants rated the contribution of 12 causes of the obesity epidemic on 7-point numeric response scales. These causes were informed by previous research [12, 21, 26–28]. Four were individual choice or behaviour (“People don’t make enough effort to exercise regularly”, “People don’t make enough effort to eat healthily”, “People don’t care about their weight”, “People don’t want to learn about healthy diets and lifestyles”), four were physical/psychological/biological limitations (“People inherit genes that contribute to weight gain”, “People are addicted to food”, “People have hormonal disruptions that contribute to weight gain”, “People have physical limitations that mean they are not able to exercise”), and four were societal/obesogenic environment (“Portion sizes in restaurants, fast-food shops, cafés and pubs are large”, “Unhealthy foods are heavily advertised, cheap, and widely available”, “Lack of time or money means people can’t exercise”, “Neighbourhoods are designed for travel by car rather than walking or cycling”). Participants were shown the implied ranking of their ratings and could confirm or re-order.

#### Blame and responsibility

Participants were asked how much they believed i) individuals, private businesses and government are to blame for the obesity epidemic in their country, and ii) how much responsibility each has to tackle it. Three sliders were used to assign a percentage of blame and responsibility for each. The total had to be 100%. This technique requires participants to make direct comparisons, avoiding ceiling and floor effects [29].

#### Policy perceptions

Participants rated six types of policies designed to tackle the obesity epidemic on fairness, effectiveness, and support on 7-point numeric response scales (see Supplementary Material). They were then shown the policies ranked by i) how much they reported supporting them and ii) how effective they thought they would be. They could confirm or change their ordering. The policy types were based on the Nuffield ladder of intervention that defines policies by their level of intrusiveness [30]. We adapted it by collapsing some categories to accommodate overlaps between levels.

#### Estimates of obesity

We designed a 10 × 10 grid of figures representing all the adults/children in the participant’s country. Participants estimated i) how many adults out of 100 have overweight, ii) how many have obesity, iii) how many children out of 100 have overweight, and iv) how many have obesity. We used natural frequencies because they are easier to understand and more accurately processed, particularly for those with lower numeracy [31–34].

#### Perceived problem

Participants rated how much they thought obesity was a public health problem on a scale from 1 to 7.

#### Mortality beliefs

We designed a ranking task that required participants to rank the mortality rate of six different causes of death (“Smoking”, “Obesity”, “Alcoholism”, “Suicide”, “Drug addiction”, “Road accidents”) from highest to lowest in their country. For analysis, we calculated the inverse of the ranking (i.e. the highest ranking scored 6, the lowest scored 1).

#### Obesity consequences

Participants were shown a list of 16 health problems and asked to select which, if any, were more likely to occur with obesity. Eight had established links to obesity (e.g., “high blood pressure”, “fertility problems”, “joint problems”), and eight did not (e.g., “Lyme disease”, “appendicitis”, “vertigo”). For analysis, participants were assigned a score out of 16 for each correct response (i.e. correctly selecting and correctly not selecting).

Finally, we collected socio-demographic information, including age, gender, educational attainment, country (Ireland, UK, US), employment status, job sector (health, government, hospitality sector, none), social class, whether participants had a child under the age of 18, weight and height.

#### Quality checks

As an attention check, participants were asked “Earlier we showed you a picture of 100 people and asked you to click on it. What did we ask you to do?”. To answer correctly participants needed both to pay attention to the current question and to have paid sufficient attention to the earlier question on rates of obesity. 98% answered correctly. We asked age twice, once in which participants typed their age into a box and once in which they selected their age category from a list. Over 99% of participants answered consistently. We also we had two forced-choice questions at the end that asked participants for any comments and any reason why their data could not be used. We screened these for meaningless responses and found none. We also checked for straight liners, speed readers and automated bots. A detailed description of all quality checks can be found in supplementary material. Aside from our own checks, we used online panel providers who carry out their own quality checks including unique identifiers, adequate payment and not oversampling panellists. In sum, the quality checks gave us confidence that the data was of high quality.

## Results

We describe differences in responses by country, and differences between experts and the public

### Did the experts and public differ in perceived causes of obesity?

In open text responses, participants gave an average of 3.2 (SD = 1.8) causes of the obesity epidemic. Causes that were coded as societal/obesogenic environment included responses such as “much of the food at grocery stores is heavily processed” and “our food industry favors low quality processed foods”; those coded as individual choice behaviour included responses such as “people are lazy” and “people make poor food choices”; those coded as physical/psychological/biological limitations were responses such as “mental health issues”, “non nutritious food is addictive” and “family genes”. Just over 50% of participants in Ireland gave a societal/obesogenic environment cause, compared to 64% of UK participants and over 70% of US participants. Nearly 90% (45/51) of the expert sample gave at least one societal/obesogenic cause of obesity.^3^

**Fig. 1:**
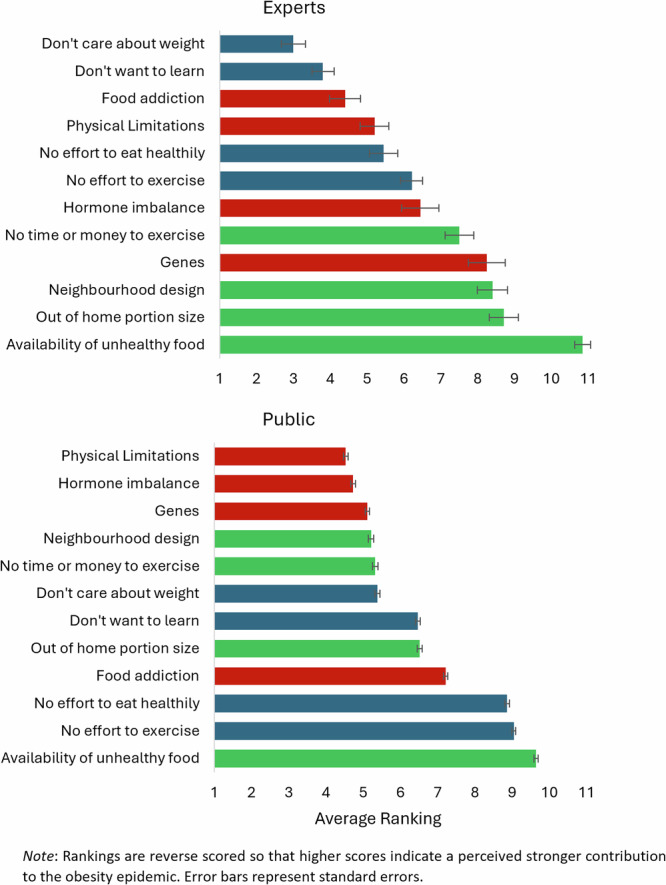
Average ranking of 12 perceived causes of obesity. The first panel shows the average ranking of causes given by the expert sample. The second shows the average ranking given by the public samples.

A similar difference between the public and expert samples emerged when participants ranked 12 causes (Fig. 1). The public ranked an environmental cause, “the availability of unhealthy food”, as the top contributor, but the second and third highest ranked causes were individual. In contrast, the top three contributors according to the expert sample were environmental.

**Fig. 2:**
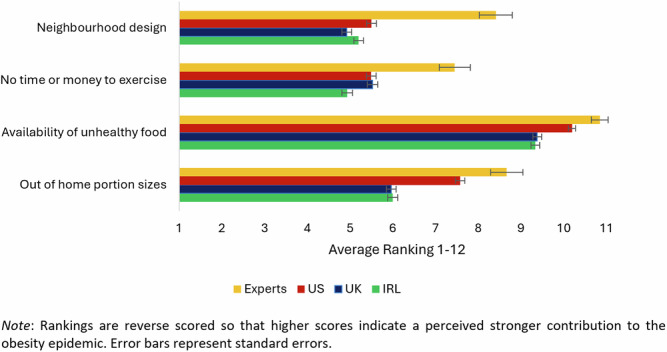
Average ranking of 4 societal causes of obesity. The average ranking of 4 societal causes of obesity given by the expert sample and by each of the 3 public samples in Ireland, the US and the UK.

Figure 2 shows the average ranking given to the four environmental causes. The expert sample ranked all four higher than the public. In keeping with the open text responses, the public in the US ranked environmental causes higher than the public in Ireland or the UK.

Table 2 shows regression analyses that test these differences. The models confirm differences between countries in perceived causes of the obesity epidemic. The experts were much more likely than the public to list and rank environmental factors – expert status had the largest coefficient of any effect.

**Table 2:** Regression models showing effects of country and expert status on perceptions of environmental causes of the obesity epidemic.

	Generated environmental cause		Rank given environmental causes	
	Model 1 - Logit	Model 2 - Logit	Model 3 - OLS	Model 4 - OLS
	Log Odds (SE)	Log Odds (SE)	B (SE)	B (SE)
Country (Ref. Ireland)				
UK	0.39 (0.10)		−0.00 (0.09)	
	*p* < 0.001		*p* = 0.990	
US	0.68 (0.11)		0.64 (0.09)	
	*p* < 0.001		*p* < 0.001	
Age	−0.02 (0.00)		−0.01 (0.00)	
	*p* < 0.001		*p* < 0.001	
Male	−0.44 (0.09)		−0.55 (0.07)	
	*p* < 0.001		*p* < 0.001	
Degree +	0.12 (0.09)		0.31 (0.08)	
	*p* = 0.216		*p* < 0.001	
Social grade (Ref. ABC1)				
C2DE	−0.21 (0.09)		−0.17 (0.08)	
	*p* = 0.031		*p* = 0.025	
Farmer/Unsure	−0.02 (0.25)		−0.06 (0.19)	
	*p* = 0.935		*p* = 0.739	
Experts (Ref. Public)		1.50 (0.44)		2.17 (0.25)
		*p* < 0.001		*p* < 0.001
Constant	1.24 (0.19)	0.51 (0.04)	7.04 (0.15)	6.67 (0.04)
	*p* < 0.001	*p* < 0.001	*p* < 0.001	*p* < 0.001
Observations	2396	2451	2396	2451

We ran Models 3 and 4 using an ordinal logistic regression and found the same effects.

### Did the experts and public differ in blame and responsibility?

**Fig. 3:**
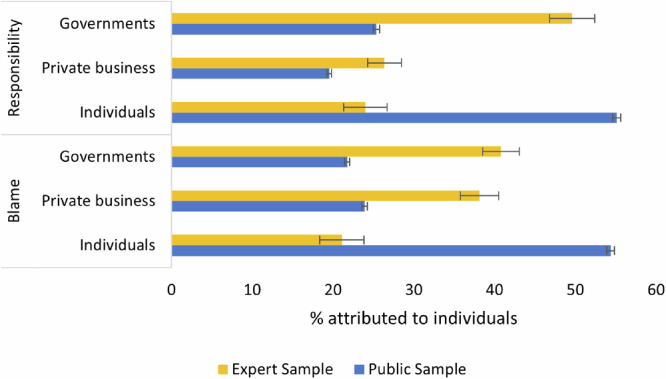
Percentage of blame for the obesity epidemic and responsibility to tackle it. The first panel shows the percentage of responsibilty assigned to governments, private business and individuals by the expert sample and by the public sample. The second panel shows the percentage of blame assigned to each by the expert sample and the public sample.

The public in all three countries apportioned the largest share of blame for the obesity epidemic to individuals over businesses and government, Fig. 3. The expert sample attributed less blame (M = 21.1%, SD = 19.6) and responsibility (M = 24.0%, SD = 19.2) to individuals than the public sample (54.4% SD = 23.9, and M = 55.1%, SD = 24.6, respectively), Models 2 and 4, Table 3. Again, the effect of being in the expert sample was by far the largest. We found no substantial country differences in assigning blame or responsibility.

**Table 3:** Linear regression models showing the relationship between country and expert status and percentage of blame and responsibility for the epidemic assigned to individuals.

	Blame	Responsibility		
	Model 1	Model 2	Model 3	Model 4
	B (SE)	B (SE)	B (SE)	B (SE)
Country (Ref. Ireland)				
UK	−0.21 (1.19)		−0.63 (1.22)	
	*p* = 0.861		*p* = 0.657	
US	−0.85 (1.23)		2.91 (1.27)	
	*p* = 0.492		*p* = 0.022	
Age	0.26 (0.03)		0.31 (0.03)	
	*p* < 0.001		*p* < 0.001	
Male	1.74 (0.97)		1.30 (0.99)	
	*p* = 0.073		*p* = 0.191	
Degree+	−1.94 (1.06)		−2.18 (1.09)	
	*p* = 0.067		*p* = 0.045	
Social grade (Ref. ABC1)				
C2DE	−1.19 (1.07)		0.45 (1.11)	
	*p* = 0.265		*p* = 0.683	
Farmer/Unsure	−2.26 (2.66)		−4.57 (2.74)	
	*p* = 0.392		*p* = 0.095	
Sector (ref. None of these)				
Hospitality	−0.66 (2.66)		−2.17 (2.74)	
	*p* = 0.804		*p* = 0.683	
Healthcare	−0.64 (1.92)		1.83 (1.97)	
	*p* = 0.737		*p* = 0.335	
Government	1.23 (2.34)		0.27 (2.41)	
	*p* = 0.601		*p* = 0.909	
Experts (ref. Public)		−33.29 (3.36)		−31.09 (3.47)
		*p* < 0.001		*p* < 0.001
Constant	42.72 (2.06)	54.37 (0.49)	39.85 (2.12)	55.11 (0.50)
	*p* < 0.001	*p* < 0.001	*p* < 0.001	*p* < 0.001
Observations	2396	2451	2396	2451

We ran the models using ordinal logistic regression and found the same effects.

### Obesity as a problem

Most participants perceived obesity to be a major health problem, Supplementary Fig. S1. The mean score in the full public sample was 6 (SD = 1.14) on a scale of 1–7. Participants in the US perceived it to be a slightly bigger problem (M = 6.4, SD = 0.9) than participants in the UK (M = 5.9, SD = 1.1) and in Ireland (M = 5.9, SD = 1.3), Model 1, Supplementary Table S1. The expert sample perceived it to be a greater problem again (M = 6.7, SD = 0.6), Model 2, Supplementary Table S1.

### Knowledge of obesity

Supplementary Fig. S2 shows that both public and expert samples produced accurate estimates (on average) of overweight and obesity in their country, compared with figures from national health surveys.

The public were also generally accurate in identifying consequences of obesity, identifying 14 consequences (and non-consequences) correctly out of 16 (Supplementary Fig. S3).

We also asked the public and expert samples to rank six different causes by mortality rate in their country. The experts gave obesity (5.35) and smoking (5.00) higher mortality ranks compared to the public (4.06 and 4.03, respectively). However, both perceived obesity and smoking to have higher mortality rates than alcohol, drugs, road deaths and suicide, Supplementary Fig. S4.

### Did the experts and public differ in their perceived fairness, effectiveness and support for policy?

**Fig. 4:**
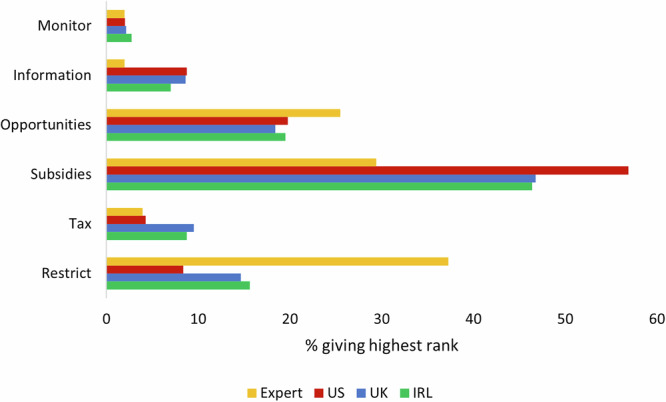
Support for each policy type. The percentage of each sample that rated each type of policy most highly.

Participants ranked support for six different types of policy that ranged in intrusiveness from monitoring obesity without intervening (lowest level) to implementing restrictions on foods or modes of transport (highest level). We compared the intrusiveness level (1–6) of the policy ranked highest on support by the public and expert samples. The experts generally endorsed more intrusive policies than the public sample, Model 2, Table 4. The public sample most strongly favoured subsidies, while the expert sample favoured restrictions, Fig. 4. The expert sample also ranked restrictions and taxes higher on effectiveness than the public, Supplementary Fig. S5. We found some country differences in policy support. Model 1 in Table 4 shows that the US sample (M = 3.78, SD = 1.02) endorsed less intrusive polices compared to the Irish sample (M = 3.98, SD = 1.03), which closely matched the UK sample (M = 3.97, SD = 1.18). Participants in Ireland and the UK were more likely to most favour restrictions and taxes than participants in the US, Fig. 4. When participants’ belief in environmental causes of obesity (i.e. the average ranking they have to environmental causes of obesity) and the responsibility they assign to individuals are added to the model, those placing more responsibility on individuals express less support for restrictive policies (Model 3, Table 4).

**Table 4:** OLS models on intrusiveness level (1–6) of the top supported policy by country and expert status.

	Model 1	Model 2	Model 3
	B (SE)	B (SE)	B (SE)
Country (Ref. Ireland)			
UK	−0.01 (0.06)		−0.01 (0.06)
	*p* = 0.903		*p* = 0.871
US	−0.17 (0.06)		−0.17 (0.06)
	*p* = 0.003		*p* = 0.004
Age	0.001 (0.001)		0.002 (0.001)
	*p* = 0.441		*p* = 0.113
Male	0.02 (0.05)		0.03 (0.05)
	*p* = 0.720		*p* = 0.584
Degree+	−0.12 (0.05)		−0.13 (0.05)
	*p* = 0.022		*p* = 0.013
Social grade (Ref. ABC1)			
C2DE	−0.01 (0.05)		−0.01 (0.05)
	*p* = 0.885		*p* = 0.929
Farmer/Unsure	0.17 (0.13)		0.16 (0.13)
	*p* = 0.181		*p* = 0.225
Sector (ref. None of these)			
Hospitality	0.02 (0.13)		0.02 (0.13)
	*p* = 0.885		*p* = 0.91
Healthcare	0.01 (0.09)		0.02 (0.09)
	*p* = 0.922		*p* = 0.848
Government	0.14 (0.11)		0.14 (0.11)
	*p* = 0.208		*p* = 0.206
Experts (Ref. Public)		0.52 (0.16)	
		*p* = 0.001	
Rank given to environmental causes			0.01 (0.14)
			*p* = 0.60
Responsibility to individuals			−0.004 (0.001)
			*p* = 0.000
Intercept	3.95 (0.10)	3.91 (0.02)	4.05 (0.16)
	*p* < 0.001	*p* < 0.001	*p* < 0.001
Observations	2396	2451	2396

We ran the models using ordinal logistic regression and found the same effects.

Ratings of fairness and efficacy of different policies mirrored policy support. Relative to the public sample, the expert sample found restrictive policies to be fairer and more effective (Supplementary Fig. S5). We ran a multilevel ordinal logistic model with policy support (rating scale 1–7) as the dependent variable and policy type as an independent variable (Supplementary Table S2), together with relevant fairness and efficacy ratings and controls for, age, gender, education, social grade, sector, and country. Perceived fairness and effectiveness were strongly associated with higher policy support.

## Discussion

These findings offer a detailed picture of the mental model of obesity held by the public in three countries, benchmarked against a sample of experts. In Ireland, the UK and the US, the public believe the obesity epidemic is a major public health problem, albeit to a lesser degree than our expert sample. This result matches a previous comparison of expert and lay risk perceptions undertaken in Canada [35]. Our results show that the public understands the prevalence of obesity and its consequences. However, we recorded a large gap between our public and expert samples about the causes of the epidemic and responsibility for it. Previous reports of such a gap have been found in the UK using small-scale qualitative data [36] and convenience samples of the public and GPs in a single area [37]. Our findings add substantially to these previous results. Using nationally representative samples in three countries, compared to our expert sample, the public were less cognisant of environmental causes of obesity and less likely to rank societal factors as strong contributors when prompted. The public attributed greater blame to individuals and assigned more responsibility for tackling the epidemic to individuals than to governments or private businesses. Those assigning responsibility to individuals were less inclined to support restrictive policies. These differences were large and highly statistically significant.

What explains these differences? It does not seem to be failure to recognise obesity as a health problem or lack of knowledge about the consequences. The public samples rated obesity as a serious problem, correctly identified multiple health conditions associated with obesity and slightly overestimated obesity rates. The public may be less aware of the obesogenic environment. That US participants were more aware of environmental causes of obesity might reflect greater abundance and salience of energy-dense, low nutrition, convenience food in the US over more decades [38]. Experts in Ireland and the UK may be attuned to this, while non-experts may need it to surpass a higher threshold to recognise it. Previous work found that public awareness of obesogenic environments does not match objective measurements [39–42]. This is unlikely to be the only explanation, however, as the public ranked “availability of unhealthy food” second on the list of possible contributors to the epidemic. The public thus differ from experts mainly in magnitude; they recognise some environmental causes of obesity, just not as strongly. Exposure to research evidence may be a contributing factor.

How important are these differences? We noted above how interventionist policies to counter obesity often face public opposition [8–10]. Our results show that the public prefer subsidies over restrictions that experts think will be effective. This is consistent with work from the UK that finds taxation and other more interventionist pricing policies are less supported than policies that promote healthier foods [43, 44]. Similar to previous findings, our public sample do support policies they think are effective and fair, but differ from the expert sample over which policies will be effective [12, 45–47]. They attribute most responsibility to individuals with much less to private business and governments. Although people typically recognise at least one environmental cause of the obesity epidemic, they place much greater emphasis on individual choice and attribute over 50% of the responsibility for tackling the epidemic to individuals. Thus, the public may be underestimating the effectiveness of some policies. Communicating the efficacy of proposed policies can increase acceptability, although only some forms of communication are effective [44, 48]. Our results shed light on why merely informing people about environmental causes of obesity does not change perceptions of causes or policy [49]. They also imply that communications focused on the effects of a policy may be more important than communications stressing the seriousness of the problem. Lastly, policymakers might note that the individual choice narrative we record can increase weight stigma which, in turn, can increase risk of obesity through obesogenic pathways [50, 51].

There are some limitations to this study. First, the expert sample was a convenience sample and hence relatively small and not derived from a representative sample frame. It was drawn from two of the three countries (Ireland, UK) and contained mostly researchers (57%) rather than healthcare workers (31%) or policy practitioners (10%). Our expert measures may therefore be subject to some sampling error. However, there was less variation in the expert responses than the public ones and the reported differences between public and expert views are large, consistent and very highly statistically significant. Furthermore, given that the representative sample of the public in the US was substantially more inclined than representative samples in Ireland and the UK to perceive societal causes of the epidemic, including US experts in the expert sample would be more likely to increase than reduce the measured differences. Overall, therefore, while accepting that point-estimates for the expert sample may be afflicted by measurement error, we deem this unlikely to explain the main findings. Second, the questions focused on causes of the obesity epidemic generally and did not ask about causes for subgroups, for example children, for which there may be different perceptions. Third, we collected data from broadly nationally representative samples based on quota sampling in all three countries, but the study was conducted online and so may have under-represented marginalised groups less likely to complete online surveys. We included controls for age, gender, education and social grade in all models to account for differences. Fourth, while we covered some biological causes of obesity, we have not discussed them in any detail. The purpose of this study is to inform public policy and as such we focus on the societal causes of obesity that policies are most likely to influence. Lastly, the study took no account of recent developments in drug therapies for obesity, which may alter perceptions of appropriate policy responses to the epidemic.

## Conclusion

This study captures how the public in three countries perceive the causes, consequences, responsibilities, blame and policy interventions for the obesity epidemic, highlighting differences between public and expert views. It is the first study, to our knowledge, to carry out such an in-depth exploration of the mental model of obesity and to benchmark against expert responses. We identify differences between public and expert samples not only in causes, but also who they see as being responsible for tackling it and the types of policies they support. Many public health communications on obesity stress the high rates of obesity and severity of consequences, but we show that the public understand these [52–54]; the public differ from experts in other areas. Improving understanding of the mental model people hold about obesity could help to design more effective communications to bring public perceptions closer to those of experts.

## Supplementary information

Supplemental Material

## Data Availability

The dataset generated during and analysed during the current study are available in the Open Science Framework repository, https://osf.io/56tmd/overview?view_only=769cba8dca59438f8899457888a92324.

## Footnotes

1 https://osf.io/56tmd/overview?view_only=769cba8dca59438f8899457888a92324

2 Ireland: RED-C, UK: Yonder Data Analytics.

3 Of the 6 experts who did not give a societal/obesogenic cause, 3 gave causes that had to be coded as ambiguous, 1 gave only a biological cause, 1 gave only an individual cause and 1 did not give any cause.
