# Supplementary material for: A mental model of obesity: comparing expert and public perceptions in Ireland, the UK, and the US
Source: Int J Obes (Lond). 2026 Jun 5;50(8):1776–83. doi: 10.1038/s41366-026-02098-z (PMC13461355; doi:10.1038/s41366-026-02098-z)
Supplement: Supplementary file 1 [file 41366_2026_2098_MOESM1_ESM.docx]

**Supplementary Material**

**A copy of the full questionnaire and data can be found on the OSF page:**	[**https://osf.io/56tmd/overview?view_only=769cba8dca59438f8899457888a92324**](https://osf.io/56tmd/overview?view_only=769cba8dca59438f8899457888a92324)

**Quality Checks**

We checked the quality of the data using four questions within the survey. The first was an attention check that required participants to remember a previous question and pay attention to the current question, that is listed in the manuscript. 98% of participants got this correct. Secondly, we asked age twice; once using an open text box in which participants typed their age and a second question in which they selected their age category from a list. We checked that these responses were consistent with each other, which over 99% were. Of the 6 people who gave inconsistent responses, 3 were within 1 year of the age bracket they chose (70 in the 60-69 age category and 30 in the < 30 age category). Finally, we had two forced choice questions at the end of the survey that asked participants to use an open text box to report any problems they had during the survey and if there was any reason their data would not be usable. All participants responded meaningfully and none said there was a reason their data could not be used.

We also checked for automated bots, straight liners and speed readers. We checked for automated bots by screening data we had collected on the timestamp of when each participant began the survey, the type of device, the browser and the screen size. We screened for duplicate timestamps that could be a sign of automated bots taking part multiple times using different accounts. Where we found the same timestamp for two participants, we checked if device type, browser or screen size were also duplicates. None were. We also checked for straight liners in the five sets of questions in the survey that it would have been possible to straight line (i.e. to give the same response to all questions). These were the ratings for causes of obesity, the ratings for support, perceived effectiveness and perceived fairness of different policies and the three questions on obesity as a public health problem. We identified 15 participants who gave the same answers in a row in each of the 5 sections. Due to the nature of the questions, however, such as how much participants would support a policy, it is difficult to determine whether straight lining to these questions was a legitimate response or not. We therefore checked their responses to our open text questions and found that all gave legitimate responses to them. We checked if the straight liners also failed the attention check and found that only 4 of them did. Finally, we had the completion time for 9 sections of the survey so we use this to check for speed readers. We created a sum of the time to complete each section and graphed the distribution, which showed a heavily right tailed skew, indicating that outliers were those who spent a longer time doing the task rather than speeding through it. As we did not identify any participant who failed all of our checks, we are confident in the quality of the data collected. In addition, aside from our own checks on the data, we only used online panel providers (RedC for the sample in Ireland, Yonder for the sample in the UK and Prolific for the sample in the US) who carry out their own quality checks on their panel including unique identifiers, adequate payment and not oversampling participants.

**Policy Question**

The wording of policy types shown to participants were as follows:

Restricting: “Policies that put limits on some unhealthy foods and inactivity.”

Taxing: “Policies that increase costs for some unhealthy foods and inactivity.”

Subsidising: “Policies that decrease costs for healthy foods and physical activity.”

Providing opportunities: “Policies that encourage and allow people to eat healthier and be more physically active.”

Informing: “Policies that give people information about food and physical activity.”

Monitoring: “Policies that collect data on the diets and physical activity levels of the population.”

Participants were given multiple examples of policies within each policy type but were asked to judge the type of policy as a whole rather than the specific examples.

**Figure S1.** Ratings of the extent to which obesity is perceived a major healthcare problem in the participant’s country.

**Figure S2.** Public and expert estimations of obesity rates in their country.

**Figure S3.** Public knowledge of consequences of obesity.

**Figure S4.** Public and expert ranking of mortality rates.

**Figure S5.** Expert and public ratings of fairness and effectiveness of different types of policy.

Table S1. Linear regression models on effect of country and expert status on perception of obesity as a public health problem (1-7).

|  | Model 1. | Model 2. |
| --- | --- | --- |
|  | B (SE) | B (SE) |
| Country (Ref. Ireland) |  |  |
| UK | -0.01 (0.09) |  |
|  | p=.899 |  |
| US | 0.94 (0.10) |  |
|  | p<.001 |  |
| Age | 0.01 (0.00) |  |
|  | p<.001 |  |
| Male | -0.11 (0.08) |  |
|  | p=.146 |  |
| Degree + | 0.08 (0.08) |  |
|  | p=.327 |  |
| Social Grade (Ref. ABC1) |  |  |
| C2DE | -0.07 (0.09) |  |
|  | p=.398 |  |
| Farmer/ Unsure | 0.18 (0.23) |  |
|  | p=.449 |  |
| Experts (Ref. Public) |  | 1.16 (0.31) |
|  |  | p<.001 |
| Observations | 2396 | 2451 |

Table S2. Multilevel ordinal logistic regression showing effect of perceived effectiveness and fairness on policy support

|  | Model 1. Level of support for any policy.  B (SE) | Model 2. Level of support for any policy.  B (SE) |
| --- | --- | --- |
| Policy Type (ref. Monitor) |  |  |
| Inform | 0.43 (0.06), p<.001 | 0.44 (0.06), p<.001 |
| Opportunity | 0.52 (0.07), p<.001 | 0.52 (0.06), p<.001 |
| Subsidies | 0.56 (0.07), p<.001 | 0.56 (0.07), p<.001 |
| Tax | 0.13 (0.06), p=.03 | 0.13 (0.06), p=.03 |
| Restrict | 0.11 (0.06), p=.06 | 0.11 (0.06), p=.06 |
| Perceived efficacy of policy | 0.95 (0.02), p<.001 | 0.96 (0.02), p<.001 |
| Perceived fairness of policy | 1.83 (0.02), p<.001 | 1.82 (0.02), p<.001 |
| Age (ref. < 30) |  |  |
| 30-39 | 0.12 (0.10), p=.22 |  |
| 40-49 | 0.11 (0.10), p=.27 |  |
| 50-59 | 0.24 (0.10), p=.02 |  |
| 60-69 | 0.19 (0.10), p=.06 |  |
| 70+ | 0.40 (0.12), p=.001 |  |
| Male | -0.09 (0.06), p=.11 |  |
| Degree+ | 0.03 (0.07), p=.65 |  |
| Social Grade (ref. ABC1) |  |  |
| C2DE | -0.05 (0.07), p=.49 |  |
| Farmer/Unsure | -0.04 (0.17), p=.79 |  |
| Sector |  |  |
| Hospitality | -0.05 (0.16), p=.76 |  |
| Healthcare | 0.14 (0.12), p=.21 |  |
| Government | 0.19 (0.14), p=.18 |  |
| Country (ref. Ireland) |  |  |
| UK | -0.26 (0.07), p<.001 |  |
| US | -0.26 (0.08), p=.001 |  |
| Experts (ref. Public) |  | 1.57 (0.21), p<.001 |
| Individual Effect | 1.25 (0.07) | 1.32 (0.07) |
